# Predicting life expectancy with a long short-term memory recurrent neural network using electronic medical records

**DOI:** 10.1186/s12911-019-0775-2

**Published:** 2019-02-28

**Authors:** Merijn Beeksma, Suzan Verberne, Antal van den Bosch, Enny Das, Iris Hendrickx, Stef Groenewoud

**Affiliations:** 1 Centre for Language Studies, Radboud University, Erasmusplein 1, 6525 HT Nijmegen, The Netherlands; 2 Leiden Institute for Advanced Computer Sciences, Leiden University, Niels Bohrweg 1, 2333 CA Leiden, The Netherlands; 3 KNAW Meertens Institute, Oudezijds Achterburgwal 185, 1012 DK Amsterdam, The Netherlands; 4 IQ Healthcare, Radboudumc, Mailbox 9101, 6500 HB Nijmegen, The Netherlands

**Keywords:** Life expectancy prediction, Advance care planning, Long short-term memory, Clinical free-text

## Abstract

**Background:**

Life expectancy is one of the most important factors in end-of-life decision making. Good prognostication for example helps to determine the course of treatment and helps to anticipate the procurement of health care services and facilities, or more broadly: facilitates Advance Care Planning. Advance Care Planning improves the quality of the final phase of life by stimulating doctors to explore the preferences for end-of-life care with their patients, and people close to the patients. Physicians, however, tend to overestimate life expectancy, and miss the window of opportunity to initiate Advance Care Planning. This research tests the potential of using machine learning and natural language processing techniques for predicting life expectancy from electronic medical records.

**Methods:**

We approached the task of predicting life expectancy as a supervised machine learning task. We trained and tested a long short-term memory recurrent neural network on the medical records of deceased patients. We developed the model with a ten-fold cross-validation procedure, and evaluated its performance on a held-out set of test data. We compared the performance of a model which does not use text features (baseline model) to the performance of a model which uses features extracted from the free texts of the medical records (keyword model), and to doctors’ performance on a similar task as described in scientific literature.

**Results:**

Both doctors and the baseline model were correct in 20% of the cases, taking a margin of 33% around the actual life expectancy as the target. The keyword model, in comparison, attained an accuracy of 29% with its prognoses. While doctors overestimated life expectancy in 63% of the incorrect prognoses, which harms anticipation to appropriate end-of-life care, the keyword model overestimated life expectancy in only 31% of the incorrect prognoses.

**Conclusions:**

Prognostication of life expectancy is difficult for humans. Our research shows that machine learning and natural language processing techniques offer a feasible and promising approach to predicting life expectancy. The research has potential for real-life applications, such as supporting timely recognition of the right moment to start Advance Care Planning.

## Background

### Introduction

Life expectancy plays an important role when decisions about the final phase of life need to be made. Good prognostication for example helps to determine the course of treatment and helps to anticipate the procurement of health care services and facilities, or more broadly: facilitates Advance Care Planning. Advance Care Planning (ACP) is the process during which patients make decisions about the health care they wish to receive in the future, in case the patient loses the capacity of making decisions or communicating about them [1]. Successful ACP enhances the quality of life and death for palliative patients, by providing timely palliative care and documenting preferences regarding resuscitation and euthanasia, among other things [1].

Accurate prognosis of life expectancy is essential for general practitioners (GPs) to decide when to introduce the topic of ACP to the patient, and it is a key determinant in end-of-life decisions [2–4]. Increasing the accuracy of prognoses has the potential to benefit patients in various ways by enabling more consistent ACP, earlier and better anticipation on palliative needs, and preventing excessive treatment. This study focuses on automatic life expectancy prediction based on medical records.

Although medical records are increasingly available in the form of electronic medical records (EMRs), they remain underutilized for developing clinical decision support systems, and improving health care in general [5, 6]. EMRs are characterized by irregularly-sampled time-series data, missing values, long-term dependencies involving symptoms, diagnoses and interventions, and are prone to documentation errors [7]. Moreover, they contain important information in the form of unstructured, textual data, from which information cannot be extracted straightforwardly. These challenges lead to suboptimal use and even waste of large portions of data [5], especially when the data is unstructured and noisy. Free texts make up a significant and important part of EMR data, but their ambiguous and noisy character and the and lack of canonical forms for medical concepts and the relations between them make it difficult to ‘mine’ these texts effectively [8].

### Prognostication: A difficult task

Accurate prognosis is notoriously difficult; a systematic review investigating the accuracy of clinicians’ estimates of survival of palliative patients shows that there is wide variation in the accuracy of predictions. Although there is a variety of tools available for identifying palliative patients, such as RADPAC [9], SPICT [10], and the Surprise Question [11, 12], virtually none of them are widely used, because using them is time-consuming, and psychological or social factors tend to be marginalized in these tools, although they are important when making end-of-life decisions [13]. In practice, the most important indicators used by GPs when making prognoses tend to be discharge letters from the hospital, increased need for medical care, and decreased social contacts [14].

Identification of patients in need of palliative care depends heavily on the experience of a doctor with palliative patients [15]. Christakis and Lamont [15] investigated the accuracy of doctors in a hospice setting: whenever a new patient was admitted to a participating hospice, a survey with the referring doctor was executed in order to obtain their life expectancy prediction for this patient. Allowing an error margin of 33% before and after the actual moment of death, the study showed that 20% of the life expectancy prognoses were correct. In line with the other studies discussed in [16], doctors systematically overestimated actual life expectancy [16] – their predictions were too optimistic. Being overoptimistic about life expectancy hinders proper end-of-life care: it may be the root cause of late hospice referral [15]. While experts agree that terminally ill patients should ideally receive 3 months of hospice care, patients in practice usually receive no more than 1 month [15, 17].

### Automatically processing clinical data

Machine learning, natural language processing, and data mining in general have grown to be increasingly popular methods for processing data within the medical domain. Given examples, machine learning algorithms can be trained to learn which pieces of information are important to execute a task, and which patterns are indicative for producing correct output. Machine learning and language processing techniques have been applied to a broad range of tasks, including medical decision support and decision making [18–20], automatic disease detection [21–23], automatic diagnostication [24–28], identifying the role of genes in the onset of diseases [29], adverse event detection [30], identifying interactions between drugs [31] and side-effects of drugs [32], and phenotyping [33].

Artificial neural networks are a special type of machine learning algorithms. Neural networks consist of interconnected layers of simple information processing units. They are used to model complex and non-transparent (e.g. mathematically non-linear) relationships between observational variables and corresponding output variables. Deep neural networks do not link observational variables directly to output variables, but introduce one or more hidden layers between input and output which are capable of representing complex intermediary solutions to the input-output mapping problem they are trained on.

Avati et al. [34] use a deep neural network to predict one-year mortality of patients during hospital admission, based on their EMR data, to identify patients who could benefit from palliative care. The authors formulate the task of predicting life expectancy as a binary classification problem, and extract only the structured data such as clinical codes from the medical histories. They used the data of the year leading up to the moment at which a prediction was made, and discretized the time line into four time slices, thereby giving more weight to data from more recent developments. They feed all data to a deep neural network with eighteen hidden layers to predict whether the patient would die within 12 months or not. Their results show the model reaches an average precision of 69%.^1^ Because early recall is beneficial for detecting palliative patients, the authors note that the recall from a high precision point onward is of interest: at a precision of 90%, the model achieves 34% recall ([34]:5).

Lumping the data into time slice bins and feeding these bins to the model at once helps to reduce the sparsity of the data. It also resolves the challenge of creating comparable patient representations from incomparable sequences of data for different patients, which result from irregular sampling. However, ignoring detailed sequential information in the data inevitably leads to information loss, such as a the order in which events took place, the rate of the disease progression, and whether the patient suffered from multiple diseases simultaneously. The present research therefore aimed to develop a predictive model that is aware of sequential information.

Rajkomar et al. [35] used EMRs from two hospitals to explore the use of deep neural networks in a variety of tasks: in-patient mortality, re-admission within 30 days, a hospital stay which lasts longer than 7 days, and discharge diagnoses. For one of the hospitals, free-text notes were available in addition to the structured data. To solve the problem of variable amounts of data for different patients, the authors trained three different models that handle this problem in different ways, and combined their outputs into final predictions. To overcome the problem of different documentation standards between hospitals, the authors imported the data in the Fast Healthcare Interoperability Resources (FHIR) standard. This approach however does not harmonize data between sites. Therefore, a model trained at one medical center cannot be transferred to a different medical center without further data processing.

### Long short-term memory (LSTM) models

Different approaches and algorithms have been designed to handle time-series data, including recurrent neural networks, hidden Markov models, and conditional random fields [36]. The absence of a strong memory in these models however leads to the inability to exploit long-distance interactions and correlations, which make these algorithms less suitable for learning long-distance dependencies typical of clinical data [36].

To address the challenges of time-series data, a specific type of recurrent neural network (RNN) was designed for modeling long-term dependencies: long short-term memory (LSTM) [37]. LSTMs, like regular RNNs, have a memory for copying the activation patterns of hidden layers. Iterative replications of hidden layer activations are used to process data through time: the activation pattern at time *t* is input to the network at time *t* + 1 along with the new input available at *t* + 1*.* The output per time step is therefore moderated by current and historical data. In addition to simple RNNs, LSTM units contain several gates: an input gate, an output gate, and a forget gate. These gates influence the flow of data through the model, allowing it to pass information to another time step only when it is relevant, thereby enabling the model to detect long-term dependencies and retain them as long as they need to be remembered.

LSTM models increasingly receive attention in the medical domain. An LSTM model was used for example to diagnose patients in a hospital setting based on sensor data such as blood pressure, temperature, and lab test results [24]. Similarly, an LSTM model was used to predict examination results given previous measurements [38]. DeepCare is an LSTM-based system used to infer the current illness state and to predict future medical outcomes [39]. There is also an increasing body of work using LSTMs for extracting specific information (medical events or medication names for example) from medical text such as scientific literature [40–42].

### Predicting life expectancy with an LSTM

Due to the increasing availability of EMR data and the success of LSTM models in many tasks, this research aims to determine the feasibility of LSTM models for predicting life expectancy based on EMR data. LSTM models are especially suitable to perform this task, because they are able to keep the sequential nature of the data intact and to exploit long-term dependencies – traits that simpler predictive models generally do not offer. We address the following questions:

1. How accurately can an LSTM trained on EMRs predict the time to death (in number of months)?
2. To what extent does the inclusion of features from unstructured textual data improve a prognostic model for detecting the approaching end of a patient’s life?

To our knowledge, there is no benchmark dataset available for this task, and no clear baseline system exists to compare our results to. Studies in this direction of research tend to be set in a hospital or hospice setting, tend to involve terminally ill patients, and tend to be disease-specific (and therefore to involve specialists). Although a direct comparison is therefore not possible within the scope of this study, we compared our results to the most similar study analyzed in the systematic review that was reported by [16] – the hospice study reported by [15] – to place our systems’ performance into perspective. With this comparison, we aimed to shed light on our final question:

## How does the prognostic accuracy of the models compare to doctors’ prognostic accuracy?

In the following sections, we describe the methods we used for training and testing the model, present and discuss results, and describe ideas for future work.

## Method

### Overview

We define the task to solve as follows: *predict the life expectancy (in number of months) of a patient at a certain moment in time, given the patient’s medical history up to that moment*. In order to learn the task automatically from data, we trained an LSTM model on medical records of deceased patients with a recorded date of death, in which the month of death functions as the target to be predicted. We optimized the model architecture and feature set, and tested the performance of several models. The following sections describe:

- the dataset;
- the train-validation-test split;
- our methods for creating the input data for the model;
- our methods for determining the model architecture;
- our methods for feature selection;
- the evaluation protocol.

### Data description

In collaboration with the academic hospital Radboudumc [43], we extracted EMRs from the FaMe-net repository [44] which stores EMRs of patients who have given consent to the use of their EMR data in scientific research. The data was collected from seven health care facilities that are part of the health care consortium of Nijmegen, the Netherlands. The dataset contains a total of roughly 33,509 EMRs. The EMRs were used as input for the model to learn which features of the data are important indicators for estimating life expectancy. For training and evaluation purposes, the model required known dates of death to function as labels. Therefore, only the pseudonymized medical records from deceased patients were included, leading to a total 1234 medical records (3.7% of the total number of patients).

The data consisted of records of 52% female patients and 48% male patients. The medical records span the five final years of life for each patient. The average age at the moment of death was 78; 81 for women and 76 for men. These averages correspond to the national averages as reported by the national data center for statistics in the Netherlands [45].

### Structured data

The EMRs contain both structured and unstructured data. Much of the information in the medical records is highly structured due to the use of standardized medical codes: ICD-10 codes (International Statistical Classification of Diseases and Related Health Problems) [46] and ICPC-1 codes (International Classification of Primary Care) [47]. ICD and ICPC codes are used to document medical information during a patient consult, such as the reason for encounter and the diagnosis. Lab tests are represented by lab codes, and lab values follow a predefined format. Labels for the type of consultation and medication come from limited sets of predefined descriptions, and are therefore well-structured as well.

### Unstructured data

In addition to structured information, EMRs contain letters sent between specialists about the patient, and notes taken during the consultation that are usually intended for personal use by the GP only. On average, 121 consultations were documented per patient for the five-year period, and for roughly 75% of the consultations, notes or letters were written. 85% of the documents are notes, and 15% are letters.

Notes and letters are free texts written in highly variable formats. Depending on whether the texts are personal notes, or meant for other readers as well, they are characterized more or less, respectively, by large amounts of noise (e.g. text formatting elements), idiosyncratic use of language, many non-standardized abbreviations, spelling errors, ungrammatical sentences, telegram-style writing and jargon.

In order to optimize and standardize the textual data for further processing, we created a typical natural language processing pipeline (a modular system in which processing subtasks are performed sequentially, passing analyses and information along) to 1) improve the quality of the texts by removing and correcting noise, 2) improve the recognition of semantically similar words, and 3) remove redundant information such as headers and footers from letters. The pipeline consists of processes to normalize the text, tokenize the text into sentences and words, add the lemmatized word form, remove headers and footers from letters, expand common abbreviations (e.g., ‘p’, ‘pt’, ‘pat’ → ‘patient’), map common synonyms to the same concept (e.g., ‘oesophagus’ / ‘esophagus’ / ‘oesofagus’ → ‘slokdarm’), provide part-of-speech tags, and correct spelling errors. For a detailed description of these processing steps and the motivation behind each, we refer the reader to [48, 49].

### Train-validation-test split

Because the number of patients per health care practice was highly variable, and to mimic real-life use of the model, we split the dataset into 90% development data (1107 patients) and 10% test data (127 patients). We set apart the 10% most recent patients from health care facilities (based on their date of death) as test data. We used the most recent patients as test data to mimic a scenario of actual deployment: if a system for automatic prognostication would be used in reality, it would be applied to new data – patients records which at no point have entered the cross-validation cycle.

We optimized the LSTM model architecture and the feature set with separate exhaustive ten-fold cross-validation procedures on the development dataset. We split the development dataset randomly into ten non-overlapping sets of 90% training data and 10% validation data for ten rounds of validation.

After tuning the hyperparameters of the model and determining the composition of the feature set, we assessed the generalization of the model by training it on all development data, and testing it on the unseen test data.

### Creating input data for the model

The LSTM model expects fixed-length input sequences, while the sequences of data points for all patients are of variable length and are characterized by irregular sampling. Therefore, we cannot simply feed the model a sequence of only the days on which a patient visited the GP: alignment with the actual time line would be lost, and sequences of different patients would not be comparable. We aggregated the data over thirty-day periods (we refer to these periods both as ‘thirty-day period’ and ‘month’ in this paper, for the sake of simplicity) to create a time line.

On average there are three consultations per patient per month, but generally only one of the three is an actual visit - others tend to be associated actions in response to a visit (e.g., administrative actions, phone calls, contacting a specialist). Therefore, we chose to aggregate data over one-month periods even though it leads to some loss of information regarding the order of events: one-month periods are large enough to solve the issues of irregular sampling and data sparsity, but small enough to capture longitudinal disease progression and to capture overall in- or decreases in the frequency of contact between the doctor and the patient.

We represented each month with one feature vector. Each vector is a frequency distribution over all features for a patient in a particular month. Each medical record in the dataset spans 5 years, and is therefore represented by 61 feature vectors, which contain frequency counts for each feature that occurred during the corresponding month.

We normalized the data per feature category, and we normalized the data per month for each patient to annul the effect of the number of consultations in a month and the length of text documents. Normalizing the data helps to prevent exploding and vanishing gradients (a common difficulty when training artificial neural networks), which would impede correct adaptation of the weights and biases of the hidden layer of the LSTM model. The frequency counts for the features were normalized to values between 0 and 1 by dividing all feature values of a feature category within a thirty-day period by the highest absolute value in that period of the patient’s history.

We want to train the model to learn to predict the life expectancy for any given moment in time. We used a sliding window to divide the complete medical history into subsequences of the history. We trained the model to the predict life expectancy for each of these subsequences, so it learns to predict the life expectancy for any given moment in the five-year time frame. The optimal window size was determined during the model optimization phase.

### Determining the model architecture

We determined the model architecture with a stepwise hyperparameter search using ten-fold cross-validation to compare various LSTM configurations, implemented with Tensorflow [50]. We experimented with the following parameters: activation function, learning rate, batch size, number of hidden layers, number of units per hidden layer, window size, peephole connections, dropout, and number of epochs.

The best performing model is a fully connected model consisting of an input layer, two hidden layers and an output layer, for each time step. We used a batch size of 5, used a learning rate of 10^− 5^, and we trained the model for ten epochs. We used the Adam Optimizer to optimize the gradient descend procedure, and used cross-entropy to minimize the loss during the training process. No dropout or peephole connections were used.

**Fig. 1:**
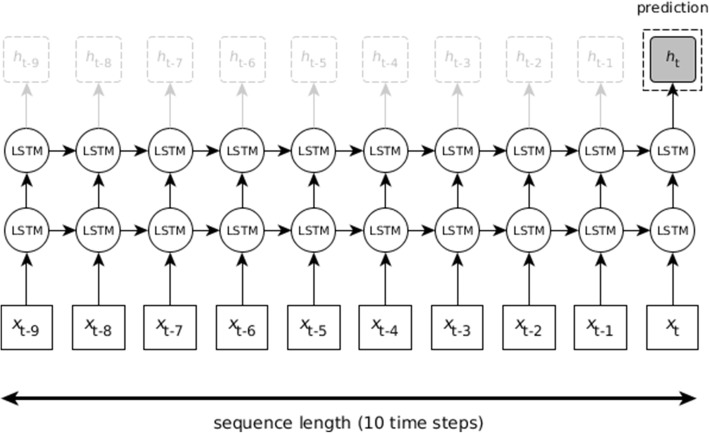
Simplified LSTM architecture. At final time step t, x_t_ represents the feature vector used as input to the hidden LSTM units, which activate output h_t_. In each preceding time step, output h functions as an intermediate prediction of life expectancy. We are interested in final prediction h_t_: a probability distribution for the next 50 months

The optimized LSTM model observes 10 time steps, or in other words, the input to the network represents a window of 10 months. For each time step, the input layer consists of a feature vector with roughly 900 to 1200 dimensions (depending on the amount of keyword features). The hidden layers contain 50 hidden units each, for which we use the tanh activation function. We initialized the weights of the hidden units randomly from a truncated normal distribution, and used a bias of 0.1. We modeled the probability that the end of life occurs at a certain moment in time by projecting life expectancy on a time line. The maximum life expectancy of the train and test cases is determined by the length of the total medical history (5 years) minus the length of the sliding window (10 months); the maximum life expectancy does not exceed this number, because predictions are made for the final time step in the window only. Therefore, the output sequence at time *t* represents a time line of 50 ‘future’ months. The model architecture is schematically illustrated in Fig. 1.

At each time step, the hidden layer is fully connected to the input and output layers of the current time step, and to the hidden layers of the previous and next time steps. Because information is passed from each time step to the next, the model considers information from all previous time steps in the window when the final prediction at the final time step is made.

**Fig. 2:**
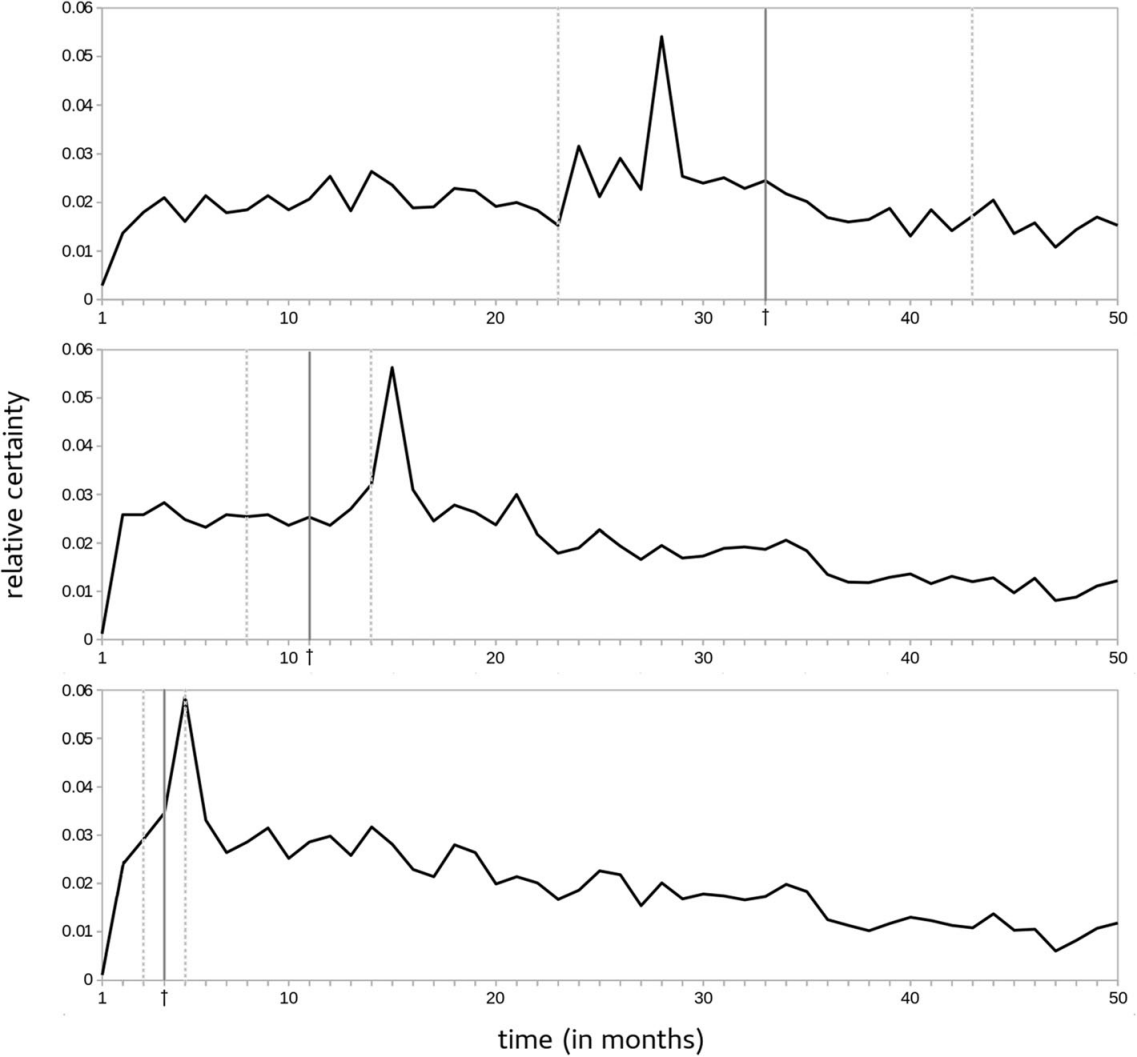
Probability distributions produced by the baseline model for one patien*t at different moments in time.* From top to bottom, the corresponding actual number of months to death are 33 months, 11 months, and 3 months, respectively

Figure 2 shows three example predictions for one patient at different moments in time. The predictions are based on three different time slices of 10 months, taken from the patient’s medical history. The model creates a probability distribution by predicting the chance that the end of life will occur during each specific month.

The output sequence is transformed by a softmax function to ensure that the probabilities for all months in the distribution together sum to 1. We interpreted the argmax of the probability distribution (the month with the highest likelihood of dying) as the life expectancy predicted by the model. In Fig. 2, the corresponding actual life expectancies at the final time step are: 33 months (predicted: 28 months), 19 months (predicted: 15 months) and 3 months (predicted: 5 months), respectively. The *y*-axis can be interpreted as a relative measure of certainty; the higher the peak, the more confident the model is about a prediction.

### Selecting features for the structured EMR data

In order to construct the feature set of the structured data, we tested several combinations of feature categories and the effect of different feature reduction methods, with the aid of an additional ten-fold cross-validation procedure. We first determined the optimal representation of the structured data by testing different frequency cut-off methods: no frequency cut-off, removal of features with an absolute occurrence < 100, removal of features with a relative frequency of < 1%, and removal of the most infrequent features that together covered 25% of the data. Additionally, we tested several levels of simplification for all ICD and ICPC codes, that have the format ‘[letter][number].[decimals]’ (e.g. D84.02, *esophageal reflux without esophagitis*). We tested abstraction to ‘[letter][number]’ (D84, *esophageal condition*), the affected system denoted with a ‘[letter]’ only (D, *the digestive system*), a broad categorization of thematic consultation elements (e.g. *standard procedure*) and a combination of the latter two (e.g. D + *standard procedure*).

The absolute occurrence cut-off boundary (< 100) yielded the best results for each feature category. The model performed best when the diagnostic ICPC codes, reasons for encounter codes, and ICD codes were simplified to codes without decimal numbers (e.g. D84.02 → D84). The codes for medical history and intervention yielded the best results when they were abstracted to a combination of the affected system and consultation element (e.g. D84.02 → D + standard procedure). Medication names were cleared from information regarding the dosage and use. Lab tests were only included when they resulted in irregular or abnormal values (as reported by the GP). These processing steps reduced the complete feature set, which included 4649 unique features, with 80% to a set of 931 features.

Finally, we wanted to exclude redundant features from the model. Testing all selections of features would have made the grid search infeasible, therefore we determined redundancy on the level of feature categories. We used a forward stepwise feature selection approach: we added the feature categories one by one in order of largest to smallest positive impact on the results; feature categories were considered to be redundant if they did not increase the model’s performance. The addition of each category led to an increase in accuracy, therefore none of the categories were considered redundant. For completeness, the order in which the feature categories were added to the feature set, was: diagnosis (ICPC), medication, ICD code, reason for encounter (ICPC), lab results, intervention (ICPC), medical history (ICPC), and consultation type.

### Selecting features for the unstructured EMR data

After applying the natural language processing pipeline to the free-text data, a large set of unique keywords remained. To reduce the dimensionality of the keyword features, we experimented with three reduction methods: 1) a frequency cut-off, for which we ordered all content words from high to low frequency and took the top *n* most frequently occurring words as features, 2) the top *n* content words with the lowest entropy score, based on the Kullback-Leibler divergence [51] between the actual frequency distribution of a word through time and an ‘optimal’ distribution, and 3) word embeddings created with word2vec. For more details about each of these keyword reduction approaches, we refer the reader to [48, 49]. The remainder of this section elaborates on the word2vec representation of the textual data.

By embedding words in a vector space, each word is represented as a point in the space by a multidimensional vector that is based on the word’s distributional properties: the contexts in which it appears in a large collection of text. Instead of using words as features, we use the dimensions of the vector space as features, and the word embeddings as feature values. Because the number of dimensions rather than the number of unique words determines the number of features, there is no need to omit keywords. Representing words with word embeddings prevents the exclusion of potentially important indicators that are possibly lost when occurrence or frequency threshold heuristics are applied.

Similar vectors indicate similar words, therefore we created document representations by calculating the mean of the feature vectors of the words in a text, which is an effective strategy for representing documents [52]. To determine the optimal model architecture and parameter settings for word2vec, we trained several word2vec models [53] with different architectures and parameter settings on the clinical texts from the EMRs (±6.000.000 words in ±150.000 texts) and subjected them to an analogy test, as described in [48].

The best-performing model made use of a skip-gram architecture, a cut-off frequency boundary of 10, a window size of 5, and 300 dimensions. We used default settings for the remaining parameters. Although the model with 300 dimensions produced the best results on the analogy task, we tested the effect of using a word2vec representation consisting of 100 and 200 dimensions as well, to control for unforeseen interaction effects with the structured data features.

We concatenated the keyword feature vector to the structured data feature vector to create a single feature vector to feed to the model. Because we could not predict how the added keyword features would interact with the structured data features that were already included in the model in terms of information overlap (e.g., the occurrence of a *word* for a certain disease may strongly correlate with the occurrence of the corresponding diagnostic *code*, thereby decreasing the added value of the new features), we created feature sets of different sizes for the frequency-based, entropy-based, and word2vec-based approaches: a small (100 added keywords), medium (200 added keywords), and large (300 added keywords) feature set.

### Evaluation protocol

We applied a third ten-fold cross-validation procedure on the development data, to test the three frequency-based, the three entropy-based, and the three word2vec-based approaches for keyword selection to see how their performance compared to a baseline model without keyword features. We compared the models’ performance in terms of root mean square error and mean deviation between the actual and the predicted life expectancy.

We selected the best-performing keyword model for each keyword selection approach, and compared these models and the baseline model to human performance. To make this comparison, we used the systematic review about doctors’ prognoses [16] to select a study comparable to ours, both in terms of the task and in terms of the outcome variable. The most comparable study was carried out in a hospice setting, and concerned a non-specific group of patients with regards to illness [15]. The doctors that participated in the research were no experts in palliative care.

Although study [15] was the most comparable study, we cannot make a direct comparison between the studies. The results reported by [15] are based on a different patient population than the results we report in this paper. In the hospice setting, 92% of the patients lived for maximally six months after admission, and the median of survival was 24 days. In our study, the maximal life expectancy was roughly four years, or fifty months. The chances of dying were evenly distributed over these months as a result of the sliding window approach, thus the median of survival was 25 months. Therefore, although life expectancy was limited in our study and not in the hospice study, patients in the hospice study had a much shorter life expectancy than in our study.

However, the task presented to the doctors in [15] and to our system was the same, and the manner in which life expectancy was expressed in both studies is comparable. In study [15], the doctors expressed their estimations on a continuous scale (e.g. in days, weeks or months), in contrast to many other studies discussed in the systematic review, which expressed life expectancy either with a limited number of predefined categories (for example, the trichotomy < 2 weeks; 2–8 weeks; > 8 weeks) or with probabilistic estimates for survival (for example the probability that the patient will live longer than three months). Due to the large number of output classes (fifty months), our outcome variable can be interpreted as a continuum, in which life expectancy is expressed in number of months to live, thereby enabling comparison to the hospice study reported in [15].

Although the significant differences between the patient population in the hospice study and our study prevent us from making a direct comparison, the similarities between the studies make a comparison informative. To provide a frame of reference, we therefore included the results of [15] in our analysis.

We adopted the evaluation criteria of the hospice-based study. The authors considered a prediction to be accurate if the actual moment of death fell within a window of 33% around the prediction. They divided the actual life expectancy by the predicted life expectancy, and regarded a prognosis as accurate if the quotient was a value between 0.67 and 1.33. Quotients smaller than 0.67 therefore signify overly optimistic errors, while values larger than 1.33 signify overly pessimistic errors [15]. By allowing a proportional deviation of 33%, the evaluation criteria are more tolerant for deviating predictions that lay further in the future than for deviations in short-term predictions.

Finally, we tested the overall best-performing model on unseen test data (consisting of the remaining 10% of the dataset), and performed additional analyses to obtain insight into the relation between predicted and actual life expectancy, and between the certainty of the predictions and life expectancy.

The following sections present:

1. the performance of the baseline model and each of the keyword models (models with a feature set including 100, 200 and 300 features for the frequency-, entropy-, and word2vec-based feature selection approaches);
2. a comparison between the performance of the baseline model and the best-performing keyword models on the one hand, and doctors’ performance in a similar task on the other hand;
3. the performance of the overall best-performing model on a held-out subset of test data;
4. additional output analyses.

## Results

### Comparing the baseline model to the keyword models

We compared the baseline model, trained on structured data only, to the keyword models in terms of the root mean square and mean deviation between the predicted and the actual life expectancy. We experimented with the number of keyword features, and the number of cells in the hidden layers, to see whether they should be increased to account for the variable amount of keyword features. In all models, the other model parameters and the set of structured data features (931 features in total) were kept constant. The results of the baseline model are shown in Table 1, and the results of several keyword models are shown in Table 2.

**Table 1:** Deviation in months between actual life expectancy and model’s predictions for the baseline model

Root mean square	Mean deviation
17.6	6.4

**Table 2:** Deviation in months between actual life expectancy and predicted life expectancy for different keyword models

Selection method	Hidden units	Root mean square deviation			Mean deviation		
		100 features	200 features	300 features	100 features	200 features	300 features
Frequency	50	17.6	17.2	17.0	4.5	5.0	5.8
	100	17.5	17.4	**16.9**	2.1	1.2	**1.7**
	200	17.7	17.8	17.8	1.6	1.3	1.0
Entropy	50	17.4	17.8	17.8	5.1	5.6	5.4
	100	17.2	**16.9**	17.8	2.5	**2.3**	1.6
	200	17.7	17.5	17.7	2.3	2.0	1.3
Word2vec	50	**17.8**	18.2	18.2	**−3.4**	−4.3	−3.7
	100	18.1	17.8	17.8	−4.2	−4.1	−4.8
	200	18.3	18.3	18.4	−3.75	−4.4	− 4.4

The models differ from each other in terms of selection method and number of included keywords. The best models are defined by two criteria: 1) having a relatively low root mean square, followed by 2) having a low mean deviation. Note: the first criterion is leading, the second criterion is only used as a tie breaker. For each selection method, the results of the best-performing model are marked with boldface, based on these criteria

As indicated with boldface in Table 2, the best-performing keyword models per selection method are:

- *model with frequency-based features*: 100 hidden units, 300 features;
- *model with entropy-based features*: 100 hidden units, 200 features;
- *model with word2vec-based features*: 50 hidden units, 100 features.

For each keyword model in Table 2, the mean deviation between actual and predicted life expectancy is lower than the mean deviation in the baseline model (as shown by Table 1). While the models (including the baseline model) tend to *overestimate* life expectancy on average, the models that include word2vec features show the opposite pattern: the negative mean deviations show that the word2vec models *underestimate* life expectancy.

### Comparing the best-performing models to doctors’ performance

We compared the results of the baseline model and the best-performing keyword model per selection method to the accuracy achieved by doctors in the hospice study [15], to get an indication of the quality of the models’ predictions.

Prognoses were considered correct if the estimation fell within a 33% window before and after the actual moment of death. According to the metric we adopted from the hospice study, the doctors’ estimates were accurate for 20% of the patients, overly optimistic in 63% of the cases, and overly pessimistic in 17% of the cases [15], as is summarized by Table 3. For the baseline model and the three best performing models that include keyword features, we evaluated the quality of the predictions with the same criteria. Table 3 shows the results of the predictions made by the baseline model and by the three models that include keyword features, in addition to the doctors’ predictions.

**Table 3:** Evaluation of the quality of the predictions

Assessor	Accuracy	Overly pessimistic	Overly optimistic
Human *EMR data + patient consultation*	20%	17%	63%
Baseline model *structured data features*	23%	58%	20%
Frequency model *structured data features + frequency-based features (keywords)*	29%	27%	44%
Entropy model *structured data features + entropy-based features (keywords)*	28%	46%	27%
Word2vec model *structured data features + word2vec-based features (vector space dimensions)*	38%	32%	31%

Predictions were considered accurate if they deviate less than 33% from the actual life expectancy. Results were adopted from [15]. Note: the doctors in [15] estimated life expectancy for a different group of patients than our models do in this the current research

As the results indicate, the baseline model outperforms the doctors’ estimates by 3% point when cross-validated on the development data. The models that include keyword features further enhance the performance compared to the baseline, especially the model that includes the word2vec-based features. Compared to the baseline model, the frequency model increases the performance with 6%, the entropy model with 5%, and the word2vec model with 15%.

### Performance of the best-performing model on unseen test data

Finally, we tested the baseline model and the word2vec keyword model on the unseen, held-out test set. The results for the baseline model and the (word2vec) keyword model are presented in Table 4, along with the human baseline.

**Table 4:** Evaluation of the quality of the predictions

Assessor	Accuracy	Overly pessimistic	Overly optimistic
Human *EMR data + patient consultation*	20%	17%	63%
Baseline model *structured data features*	20%	68%	12%
Keyword model *structured data features + word2vec-based features*	29%	52%	19%

Predictions were considered accurate if they deviate less than 33% from the actual life expectancy. The human results were adopted from [15]. Note: the doctors in [15] estimated life expectancy for a different group of patients than our models do in this the current research

Compared to the results presented in Table 3, the models’ accuracy for the held-out validation set drops: − 3% point for the baseline model and − 9% point for the keyword model. The unseen test set contains data that the model does not encounter in training, and while this did not seem to affect the accuracy of the baseline model much compared to the cross-validation experiments, it notably affects the performance of the keyword model. The results of the baseline model however match the quality of the predictions made by doctors precisely, and the keyword model increases the accuracy with 9% compared to the human predictions and compared to the baseline model.

### Additional output analyses

We further analyzed the results of the keyword model in terms of Pearson’s product-movement correlation coefficients, expecting to find a positive correlation between the actual and the predicted life expectancy. Additionally, we expected the model’s certainty (plotted as the *y*-axis in Fig. 2) to both increase as the *actual* moment of death approached, and as the *predicted* moment of death approached. We therefore expected to find negative correlations between the relative certainty of the predictions on the one hand, and the actual/predicted life expectancies on the other hand. Finally, we expected to find a higher level of certainty for predictions that are close to the actual life expectancies. Therefore, we expected the relation between the number of months between actual/predicted life expectancy on the one hand, and the certainty of the predictions on the other hand to be inversely proportional to each other. The tests, hypotheses, and results of the calculations are summarized in Table 5.

**Table 5:** Results for correlation calculations between several outcome measures

Tested relations	Hypotheses	Pearson’s *r*	Significance *p*
Actual vs. predicted life exp.	positive relation	.36	<.001
Certainty vs. actual life exp.	negative relation	−.35	<.001
Certainty vs. predicted life exp.	negative relation	−.61	<.001
Certainty vs. absolute difference between actual and predicted life exp.	negative relation	−.02	.12

**Fig. 3:**
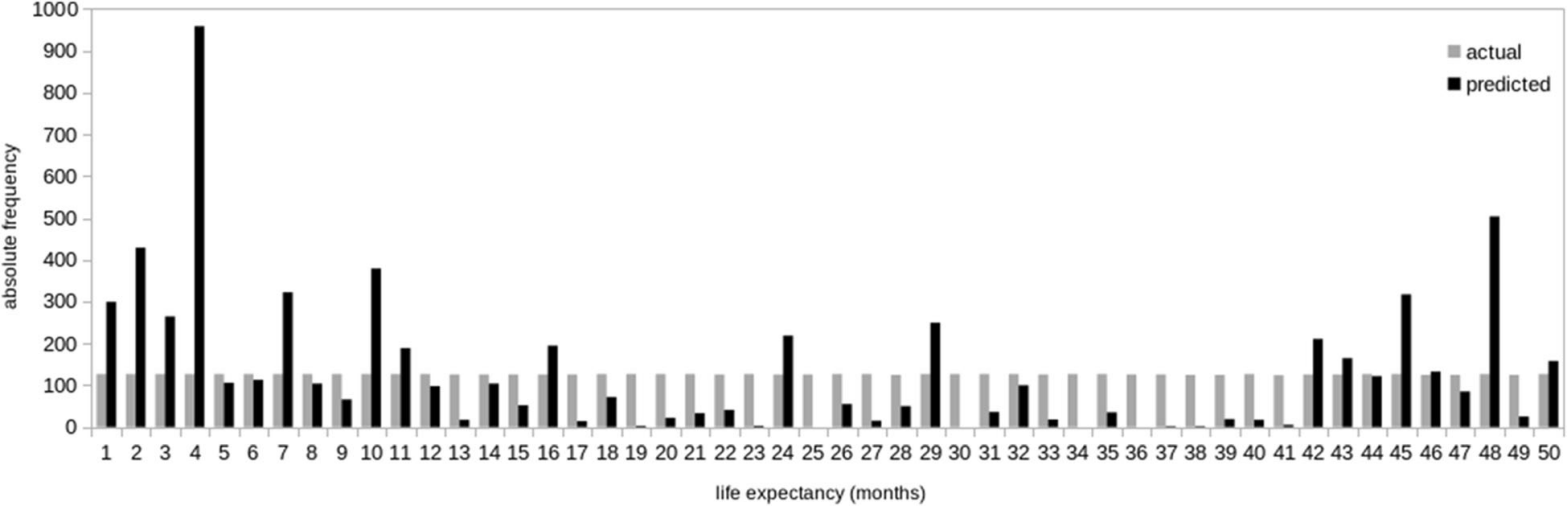
Absolute frequency counts for actual and predicted life expectancies, for each month in range 1–50

As Table 5 shows, the calculations confirmed most of the hypotheses. The results show a moderately positive relation between the model’s predictions and the actual life expectancy. To zoom in on the relation between actual and predicted life expectancy, Fig. 3 shows frequency counts of actual and predicted life expectancies. The actual life expectancies are uniformly distributed: because the medical histories are divided in 10-month windows, every month in the range 1–50 is predicted 127 times, corresponding to the 127 test patients. The predictions are not as evenly distributed as the actual expectancies: the model shows a tendency to predict that the moment of death is either relatively nearby (< 1 year) or relatively far away (> 3.5 years) in time.

**Fig. 4:**
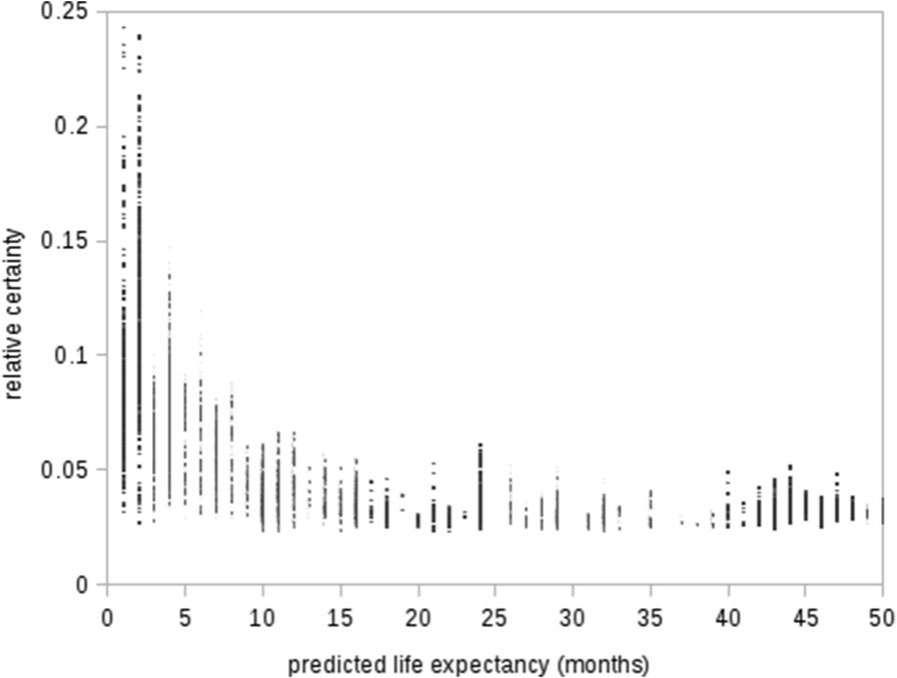
Relative certainty as a function of predicted life expectancy

The moderate negative correlation between certainty and *actual* life expectancy (*r* = −.35), and the strong negative correlation between certainty and *predicted* life expectancy (*r* = −.61) in Table 5 show the model’s tendency to be increasingly certain about predictions as life expectancy is shorter. To illustrate this tendency, Fig. 4 shows the model’s certainty as a function of the predicted life expectancy. The relative certainty with which the predictions are made is not a good indicator of the model’s accuracy however, as shown by the bottom test results in Table 5: no significant correlation exists between certainty and the absolute difference between *actual* and *predicted* life expectancy. Therefore, our expectation about a higher model certainty for more accurate predictions, was not reflected by the results.

## Discussion

### Comparison to human performance

To put the reported results in perspective, we provided a comparison of the model’s performance to human performance as described by [15]. To make a truly valid comparison, our study design should include judgments about life expectancy from GPs about the actual patients that the medical records used for this research correspond to. Making this comparison was however impossible within the scope of this research, and with the use of this dataset.

To our knowledge, no studies have been carried out in which GPs performed the task of predicting life expectancy for a non-specific group of patients. The most comparable study from the systematic review [16] concerned a non-specific group of patients in terms of illness, which was judged by clinicians from a broad spectrum of disciplines [15].

Although the study is similar to ours, there are important differences: patients were known to be terminally ill in the hospice study. Therefore, the potential life expectancy was technically not limited – death was usually rather imminent. Our dataset consisted of the medical records from the final five years of deceased patients. Life expectancy was limited to fifty months due to the sliding window approach, and the chances of dying were evenly distributed over these months. Because our study did not focus on terminally ill patients, the actual range of time to death was broader in our study, even though life expectancy was limited.

However, as prognostic accuracy tends to be inversely related to a longer life expectancy [16, 54, 55], we assume that the task we formulated was relatively hard compared to the task presented to the doctors: because life expectancy was uniformly distributed over 1–50 months in our research, the model had to make predictions about the near future (one month into the future) as well as the far future (fifty months into the future). We contrasted our study to the hospice study [15] regardless of the differences between the two to sketch a broader background. To correct for the difference between tasks in our study and [15] at least partly, we adopted the relative error margin of 33% from [15]. To enable a perfect comparison however, the system should be presented with the same test data as doctors – an issue we intend to address in future work.

### Data limitations

One of the main challenges we faced during this research was the amount of available data. Our dataset consisted of roughly 1200 patients which is a fair amount of data according to clinical standards, but is not considered to be a lot of data for training neural networks. We partially addressed this problem by splitting each medical record into fifty time slices, thereby increasing the number of cases with a factor of fifty. However, more data would have been desirable for training the model, in order to increase the accuracy and reduce overfitting.

Overfitting is a serious issue which we did not fully manage to tackle, even though we maximized the amount of training data, used cross-validation and early stopping, and explored the effects of drop-out in the neural network. We expect that the use of more data in future research will aid in a better feature selection process, especially with regards to the textual features, and will help the model to generalize better to unseen cases. Additionally, more data would enable us to explore whether disease-specific training of the model is beneficial, for example by training the model to make predictions specific for trajectories associated with cancer, dementia, or heart failure.

### Interpretation of the output

We choose to return a probability distribution for a large range of months, rather than producing a single-value prediction or a classification with few classes. While such output indeed delivers very interesting results, we also needed a way to operationalize these probability distributions in order to evaluate the model’s performance. In this research, we considered the argmax of a distribution as the final prediction. However, this is just one of many possible approaches. Alternative methods for processing the model’s output include reporting the first, the last, or any peak above a certain probability threshold, and reporting sudden changes in life expectancy. Determining whether or not alternative output variables or interpretations of the current output variable would better suit the task of predicting life expectancy, fell outside the scope of this research, but would be interesting to take into account in future research.

### Transparency

When it comes to incorrect predictions, both the baseline and the keyword model tend to make overly pessimistic predictions. It would be interesting to investigate *why* the models have a tendency toward overly pessimistic predictions, despite being trained with and tested on balanced data.

Related to this question, is the observation that the model tends to predict that the moment of death is either relatively close or far away in time, rather than somewhere in between, again despite being trained and tested on balanced data. We could speculate that the decline in health is generally gradual over a long period of time, while the transition from good health to the onset of severe illness may be sudden, as well as the transition from illness to death. The occurrence of features that are associated with such changes, may be causing the model to overfit on those features. Further exploration of which factors were leading in a prediction, may be helpful to understand which factors aid in accurate and inaccurate predictions.

A crucial issue to address in future research therefore, is the ‘black box’ character of the model. Being aware of the reliability of a model’s predictions may be sufficient for a model to have real-life applications, but does not help us to gain insight in which (combinations of) factors determine a correct prognosis. In future work, we plan to explore methods for gaining more insight in the nature of the patterns that are detected by neural networks, as well as making the determinants of a certain prediction transparent.

## Conclusions

We aimed to advance the understanding of what is needed for automatic processing of electronic medical records, and to explore the use of unstructured clinical texts for predicting life expectancy. The potential use of automatic prognostication is not limited to health care in practice, but could also be useful in other clinical applications such, such as clinical trials. In clinical trials, outcomes often depend on prognostic factors. Automatic processing of medical records would enable quick and systematic stratification of patients based on their prognoses, which could be used to further reduce biases [56].

Our contributions to previous work are that we combine the following elements into one model: 1) in addition to using structured data fields, we investigate the use of textual features that we extracted from the unstructured, clinical free-text, 2) we retain the sequential order of the medical events through time at a month-level, 3) we express life expectancy in terms of months rather than as a classification task with a small amount of categories (such as dichotomous classes, e.g. ‘mortality is expected *within* or *after* a year’), and 4) our research focuses on primary care data (rather than hospice or hospital data) of a general patient population; we made no selection based on disease (e.g. cancer patients), department (e.g. ICU patients), age (e.g. elderly patients), or course of treatment (e.g. palliative / terminally ill patients).

Using the evaluation criteria that were used by [15] to evaluate doctors’ performance in a similar task, our baseline model reached a level of accuracy similar to human accuracy (20% accuracy). The keyword model improves the prediction accuracy with 9% point to 29% accuracy. This model tends to make rather pessimistic predictions, while doctors tend to do the opposite. Pessimistic predictions could promote early recognition and anticipation of the palliative phase, and timely discussion of ACP strategies.

Even though the model’s performance is far from perfect, we consider this work to be among the first steps in a line of research that has much potential for clinical applications, for several reasons: good prognostication has the potential to contribute significantly to end-of-life decision making, therefore we believe that any increase in prognostic accuracy is worth persuing. Additionally, human prognostication is costly, time-consuming, requires medical expertise, and is a subjective task. Without compromising prediction accuracy, the model is able to make predictions quickly, automatically and systematically, while it does not depend on human medical expertise. Even though the model reaches only 29% accuracy, we consider 9% point improvement to be promising, considering that the model is trained on a relatively small data sample.

Nevertheless, this research should be considered to be exploratory. In order to replicate and extend this research, we are currently expanding the dataset substantially, by collecting additional data of both deceased and active patients. This will allow us to zoom in on specific illness trajectories, and to rephrase the task in such a way that it will match clinical settings more closely, for example by aiming to make predictions about patients while they are still active. We plan to compare a range of predictive models, alternative patient representations, and (interpretations of) output variables in future work. To provide a better comparison between automatic and human prognostication, we will investigate the prediction accuracy of both the system and general practitioners by presenting them with the same task and test data. Additionally, we will work towards obtaining insight about the driving forces behind good prognostication. We intend to explore which information is used by the model, to make the model for automatic prognostication more transparent, and improve our understanding of this complex task.

## Abbreviations

ACP: Advance Care Planning
EMR: Electronic medical record
GP: General practitioner
ICD: International Classicifaction of Diseases
ICPC: International Classification of Primary Care
LSTM: Long short-term memory
RADPAC: Radboud indicators for palliative care needs
RNN: Recurrent neural network
SPICT: Supportive and Palliative Care Indicators Tool

## Footnotes

1 Due to the skewed distribution of the data (7% prevalence), the authors prefer to discuss their results in terms of precision and recall, rather than sensitivity and specificity, because it provides more information about the algorithm’s performance ([34]:5).
